# Changes of Percent Body Fat as a Useful Surrogate for Risk of Declined Renal Function

**DOI:** 10.1038/s41598-018-35601-2

**Published:** 2018-11-23

**Authors:** Yuan-Yuei Chen, Wen-Hui Fang, Chung-Ching Wang, Tung-Wei Kao, Yaw-Wen Chang, Hui-Fang Yang, Chen-Jung Wu, Yu-Shan Sun, Wei-Liang Chen

**Affiliations:** 1 Department of Internal Medicine, Tri-Service General Hospital Songshan Branch; and School of Medicine, National Defense Medical Center, Taipei, Taiwan Republic of China; 2 Division of Family Medicine, Department of Family and Community Medicine, Tri-Service General Hospital; and School of Medicine, National Defense Medical Center, Taipei, Taiwan Republic of China; 3 Division of Geriatric Medicine, Department of Family and Community Medicine, Tri-Service General Hospital; and School of Medicine, National Defense Medical Center, Taipei, Taiwan Republic of China; 4 Graduate Institute of Clinical Medical, College of Medicine, National Taiwan University, Taipei, Taiwan Republic of China; 5 Division of Family Medicine, Department of Community Medicine, Taoyuan Armed Forces General Hospital, Taoyuan, Taiwan Republic of China

## Abstract

The association between anthropometric indices with chronic kidney disease (CKD) was examined previously. However, the effect of body fat on renal function was not determined clearly. Our aim was to investigate the association of percent body fat (PBF) and renal function in adult population from health examination in Tri-Service General Hospital (2010–2016). 35087 participants aged 20 years and older were enrolled in the study. PBF was measured by bioelectrical impedance analysis (BIA). Estimation of renal function was performed by Taiwanese MDRD equation. Optimal cut-off values of PBF was accessed by a receiver–operator characteristic (ROC) curve analysis. Multivariate regression models were used in the relationship among changes of PBF, renal function, and future CKD. In terms of baseline PBF for CKD, optimal cut-off values of PBF in males and females were 21.55 and 40.75. The changes of PBF were more closely associated with renal function decline than waist circumference (WC) with *β* values of −0.173 (95% CI: −0.233, −0.112) and −0.077 (95% CI: −0.104, −0.049), respectively. After stratified by gender, this relationship remained significant in male population with *β* values of −0.276 (95% CI: −0.371, −0.181) and −0.159 (95% CI: −0.207, −0.112), respectively. Female subjects with increased baseline PBF over cut-off values had increased risk for predicting the future CKD with odd ratios (ORs) of 2.298 (95% CI: 1.006–5.252). Body fat had detrimental impact on renal function and development of CKD in adult population. Measurement of PBF for surveillance of renal function impairment was warranted.

## Introduction

Chronic kidney disease (CKD) was an emerging public health problem worldwide and increased incident and prevalence of end-stage renal disease (ESRD) was noted in Taiwan^1^. Impact of CKD elevated risk of all-cause mortality and cardiovascular diseases. Obesity was also a common risk factor for developing cardiovascular disease and metabolic syndrome in Taiwan^2,3^. Previous studies had reported the relationship between obesity with renal function by using different anthropometric parameters. The risk of developing incident CKD was higher in the obese defined by body mass index (BMI) than normal weight subjects^4^. In a previous study, waist-to-hip ratio (WHR) had more close association with the incident CKD and mortality rather than BMI^5^. Madero *et al*. demonstrated that visceral adipose tissue had significant association with renal function decline and had risk of developing CKD^6^.

Percent body fat (PBF) was suggested as a more valid predictor than BMI for the risk of cardiovascular diseases and other adverse outcomes^7^. In a Korean study, increased PBF was significantly associated with inflammation and decline of renal function among elderly population. However, it appeared that little research findings were available concerning the effect of PBF variation on renal function in adult population. The objective of our study was to investigate whether PBF would contribute to the change of renal function in adult population from Taiwan.

## Results

### The demographic characteristics of study sample

Characteristics of both male and female participants attended baseline examination and completed second visit were listed in Table 1. The mean age of baseline visit and second visit in males and females were 38.85 ± 14.57, 39.79 ± 14.81 and 41.10 ± 16.03, 42.16 ± 16.15 years, respectively. The values of MDRDGFR were 100.62 ± 18.31, 100.36 ± 18.50 and 108.38 ± 22.40, 108.58 ± 22.97, respectively. The values of eGFR were 102.62 ± 15.22, 102.15 ± 15.46 and 120.84 ± 16.03, 120.30 ± 16.34, respectively. The prevalence of obesity was increased in second visit that 21.6% in males and 12.0% in females. Anthropometric parameters including BMI, PBF, and waist circumference (WC) and biochemical data had significant differences across these groups. There were significantly increased PBF, WC and decreased eGFR between baseline and second visit in both genders.

**Table 1:** Characteristics of study sample before and after follow-up.

Variables	Male			Female		
	Baseline Visit (N = 18514)	Second Visit (N = 18514)	P Value	Baseline Visit (N = 16573)	Second Visit (N = 16573)	P Value
**Continuous Variables, mean (SD)**						
Age (years)	38.85 (14.57)	39.79 (14.81)	<0.001	41.10 (16.03)	42.16 (16.15)	<0.001
BMI (kg/m^2^)	24.76 (3.91)	24.86 (3.93)	<0.001	22.57 (3.96)	22.68 (4.01)	<0.001
PBF (%)	24.85 (6.40)	24.91 (6.40)	<0.001	31.85 (6.72)	31.93 (6.71)	<0.001
WC (cm)	84.21 (10.28)	84.56 (10.28)	<0.001	74.34 (10.27)	74.72 (10.34)	<0.001
MDRDGFR	100.62 (18.31)	100.36 (18.50)	<0.001	108.38 (22.40)	108.58 (22.97)	<0.001
eGFR	102.62 (15.22)	102.15 (15.46)	<0.001	120.84 (16.03)	120.30 (16.34)	<0.001
Cr	0.81 (0.17)	0.80 (0.17)	<0.001	0.81 (0.17)	0.80 (0.17)	<0.001
UA (mg/dL)	6.38 (1.31)	6.38 (1.30)	<0.001	4.71 (1.06)	4.76 (1.07)	<0.001
AST (U/L)	22.42 (14.27)	22.29 (14.53)	<0.001	18.82 (10.37)	18.91 (12.90)	<0.001
Albumin (g/dL)	4.59 (0.30)	4.55 (0.29)	<0.001	4.45 (0.30)	4.41 (0.28)	<0.001
TSH (uIU/mL)	2.10 (1.43)	2.11 (1.50)	<0.001	2.41 (1.87)	2.42 (1.88)	<0.001
hsCRP (mg/dL)	0.25 (0.56)	0.25 (0.54)	<0.001	0.21 (0.42)	0.22 (0.44)	<0.001
FPG (mg/dL)	93.75 (22.96)	94.32 (22.76)	<0.001	91.08 (19.63)	91.32 (19.75)	<0.001
HDL-C (mg/dL)	48.50 (11.64)	48.22 (11.44)	<0.001	60.36 (14.16)	59.78 (13.77)	<0.001
**Category Variables, (%)**						
Proteinuria	5244 (28.3)	4518 (27.3)	<0.001	4225 (25.5)	5043 (27.2)	<0.001
Smoking	2989 (16.1)	559 (3.4)	0.002	3138 (16.9)	526 (3.2)	0.450
HTN	2676 (14.5)	1340 (8.1)	<0.001	3128 (16.9)	1754 (10.6)	<0.001
DM	505 (3.0)	750 (4.1)	<0.001	806 (4.4)	511 (3.1)	<0.001
Obese	3706 (20.0)	4008 (21.6)	<0.001	1731 (10.4)	1985 (12.0)	<0.001

BMI, body mass index; PBF, percentage body fat; WC, waist circumference; MDRDGFR, Modification of Diet in Renal Disease Glomerular Filtration Rate; eGFR, estimated Glomerular Filtration Rate; Cr, creatinine; UA, uric acid; AST, aspartate transaminase; TSH, thyroid stimulating hormone; hsCRP, high sensitive C-reactive protein; FPG, fasting plasma glucose; HDL-C, high density lipoprotein cholesterol; HTN, hypertension; DM, diabetes mellitus.

### Association among changes of PBF, WC and changes of renal function during follow-up

In Table 2, the changes of PBF and WC had significant associations with the changes of estimated glomerular filtration rate (eGFR) during the follow-up period. After multivariable adjustment, increased PBF had more closely associated with reduced renal function than WC with β values of −0.174, −0.172 and −0.173 (95% confidence interval (CI) = −0.234, −0.114; −0.233, −0.112; −0.233, −0.112) in each model, respectively.

**Table 2:** Association among changes of PBF, WC, and changes of renal function in the period of follow-up.

Variables	Model^a^ 1 *β*^b^ (95% CI)	P Value	Model^a^ 2 *β*^b^ (95% CI)	P Value	Model^a^ 3 *β*^b^ (95% CI)	P Value
**Changes of eGFR**						
Changes of PBF	−0.174 (−0.234, −0.114)	<0.001	−0.172 (−0.233, −0.112)	<0.001	−0.173 (−0.233, −0.112)	<0.001
Changes of WC	−0.078 (−0.105, −0.050)	<0.001	−0.077 (−0.105, −0.050)	<0.001	−0.077 (−0.104, −0.049)	<0.001

^a^Adjusted covariates:

Model 1 = age + gender + BMI.

Model 2 = Model 1 + proteinuria, UA, AST, albumin, TSH, hsCRP, FPG, HDL-C.

Model 3 = Model 2 + history of smoking, HTN, DM.

Gender differences in the association among changes of PBF, WC and changes of renal function were also presented in Table 3. Both PBF and WC had negative relationship with the changes of eGFR, especially in male population. The increased β values of PBF was higher than those of WC in each adjusted model.

**Table 3:** Association among changes of PBF, WC, and changes of renal function categorized by gender.

Gender	Variables	Model^a^ 1 *β*^b^ (95% CI)	P Value	Model^a^ 2 *β*^b^ (95% CI)	P Value	Model^a^ 3 *β*^b^ (95% CI)	P Value
**Changes of eGFR**							
Male	Changes of PBF	−0.280 (−0.375, −0.186)	<0.001	−0.277 (−0.372, −0.182)	<0.001	−0.276 (−0.371, −0.181)	<0.001
	Changes of WC	−0.161 (−0.208, −0.113)	<0.001	−0.162 (−0.209, −0.114)	<0.001	−0.159 (−0.207, −0.112)	<0.001
Female	Changes of PBF	−0.022 (−0.085, 0.042)	0.503	−0.022 (−0.086, 0.042)	0.500	−0.021 (−0.085, 0.043)	0.524
	Changes of WC	−0.002 (−0.028, 0.025)	0.889	−0.001 (−0.028, 0.025)	0.926	−0.001 (−0.028, 0.025)	0.931

^a^Adjusted covariates:

Model 1 = age + BMI.

Model 2 = Model 1 + proteinuria, UA, AST, albumin, TSH, hsCRP, FPG, HDL-C.

Model 3 = Model 2 + history of smoking, HTN, DM.

### Hazard ratios for predicting the changes of renal function stratified by gender

Adjusted hazard ratios (HRs) of the changes of PBF and WC for predicting the changes of renal function in males and females were presented in Table 4. However, no significant difference was noted among the adjusted models in the changes of PBF or WC among both genders.

**Table 4:** Cox hazard proportional model for changes of PBF and WC in predicting changes of renal function.

Variables	Model^a^ 1 HR (95% CI)	P Value	Model^a^ 2 HR (95% CI)	P Value	Model^a^ 3 HR (95% CI)	P Value
**Changes of eGFR**						
**Changes of PBF**						
Total	0.968 (0.851–1.101)	0.622	0.979 (0.862–1.111)	0.741	0.980 (0.863–1.113)	0.753
Male	1.051 (0.876–1.261)	0.594	1.059 (0.888–1.264)	0.523	1.061 (0.882–1.275)	0.531
Female	0.898 (0.733–1.100)	0.299	0.890 (0.726–1.090)	0.261	0.894 (0.731–1.093)	0.274
**Changes of WC**						
Total	0.985 (0.927–1.046)	0.619	0.988 (0.928–1.051)	0.695	0.988 (0.928–1.051)	0.695
Male	0.984 (0.890–1.088)	0.758	0.999 (0.900–1.109)	0.987	1.001 (0.899–1.114)	0.990
Female	0.993 (0.914–1.078)	0.858	0.995 (0.916–1.081)	0.913	0.994 (0.915–1.079)	0.880

^a^Adjusted covariates:

Model 1 = age + gender + BMI.

Model 2 = Model 1 + proteinuria, UA, AST, albumin, TSH, hsCRP, FPG, HDL-C.

Model 3 = Model 2 + history of smoking, HTN, DM.

### Adjusted odds ratios for developing CKD stratified by gender

Because the Cox proportional hazard models did not show any significant effect of the changes of PBF and WC on renal function, we further determined gender specific cut-off values of baseline PBF for CKD. Optimal cut-off values of baseline PBF categorized by gender were assessed by using receiver–operator characteristic (ROC) curve analysis in our study (Table 5). In male population, the area under the ROC (AUROC) value was 0.531 (95% CI: 0.425–0.637) and the optimal cut-off value was 21.55 with sensitivity and specificity of 85% and 30%. In females, the AUROC value was 0.613 (95% CI: 0.547–0.680) and the optimal cut-off value was 40.75 with sensitivity and specificity of 30% and 91%.

**Table 5:** Optimal cut-off values of PBF in males and females.

	AUC (95%CI)	Sensitivity	Specificity	P-value	Cut-off values
Male	0.531 (0.425–0.637)	85%	30%	<0.001	21.55
Female	0.613 (0.547–0.680)	30%	91%	<0.001	40.75

Association between the optimal cut-off values of baseline PBF with the presence of the future CKD was shown in Table 6. Female participants with increased PBF that over cut-off values had increased risks for predicting the presence of future CKD with ORs of 2.679, 2.360 and 2.298 (95%CI = 1.203–5.964; 1.039–5.363; 1.006–5.252) in each adjusted model, respectively. There was no interaction between cut-off values of baseline PBF and the future CKD. The interaction term between these factors was not significant in all models (*P* > 0.05).

**Table 6:** Adjusted odd ratio for CKD stratified by gender specific cut-off values of PBF.

Gender	Cut-off values of PBF	Model^a^ 1 OR (95% CI)	*P* Value	Model^a^ 2 OR (95% CI)	*P* Value	Model^a^ 3 OR (95% CI)	*P* Value
CKD							
Male	21.55	0.782 (0.178–3.443)	0.745	0.662 (0.148–2.953)	0.589	0.656 (0.147–2.933)	0.581
Female	40.75	2.679 (1.203–5.964)	0.016	2.360 (1.039–5.363)	0.040	2.298 (1.006–5.252)	0.048

^a^Adjusted covariates:

Model 1 = age + gender + BMI.

Model 2 = Model 1 + proteinuria, UA, AST, albumin, TSH, hsCRP, FPG, HDL-C.

Model 3 = Model 2 + history of smoking, HTN, DM.

## Discussion

In our study, we highlighted the detrimental impact of body fat accumulation in the decline of renal function in general population derived from the longitudinal analysis of health examinations. Particularly, female participants with higher baseline PBF over cut-off values had higher risks of developing future CKD. To the best of our knowledge, the present study was the first to explore the relationship between PBF and renal function, defined by Taiwanese MDRD equation, and predict the risk of future CKD by baseline PBF in a large population-based survey which was composed of general population in Taiwan.

The interactions between obesity and renal function had been reported in previous studies. In a cross-sectional observational study, subjects with increased BMI was suggested to have increased risk of CKD^8^. Boer *et al*. demonstrated that obesity was associated with a decline in GFR in a community-based population of older adults^9^. Central body fat distribution was significantly associated with impaired renal function^10^. Increased abdominal obesity, defined by WC and WHR, was positively related to renal function impairment in Chinese population^11^. In a prospective study composed of 390 elderly participants, Oh *et al*. proposed that a change in PBF was associated with a decline in eGFR estimated by CKD-EPI equation that the highest tertile of change in PBF had increased risk for rapid progression of renal dysfunction^12^. It was similar with our findings that changes of PBF had adverse effect on renal function. However, the estimation of GFR in the present study was used by Taiwanese MDRD equation, which was more suitable for Taiwanese adults than other measurements^13^. In addition, the study sample was obtained from a large-scale general population. PBF also had predictive ability for the future CKD in female population by a longitudinal analysis.

The exact mechanisms of obesity on renal function decline was unclear. Numerous studies had reported that deteriorated renal consequences by adipose tissue might include inflammation, insulin resistance and renin-angiotensin-aldosterone system (RAAS). Various cytokines such as interleukin-6 (IL-6), IL-8, IL-10 and tumor necrosis factor-alpha (TNF-alpha) were released by adipose tissue in obese subjects^14^. Increased production and decreased clearance of pro-inflammatory cytokines was proposed to cause chronic inflammatory status in CKD^15^. Emerging evidence had considered adipose tissue as an important endocrine organ which produced adiponectin, leptin, and resistin^16–18^. These hormones could lead to insulin resistance and activate progression of renal disease by worsening renal hemodynamics by several pathways including sympathetic nervous system excitation, sodium retention and downregulation of the natriuretic peptide system^19^. The RAAS was well known for regulating blood pressure and determining target-organ damage^20^. Angiotensin II was the key factor of the RAAS to increase the glomerular hydraulic pressure and the ultrafiltration of plasma proteins predominantly by vasoconstrictor effect of post glomerular arterioles, leading to the onset and progression of chronic renal damage^21^. Adipose tissue was regarded as the source of angiotensin that a local RAAS was present in human adipose tissue^22^. Besides, increased angiotensinogen produced by adipose tissue might be responsible in part for the metabolic and inflammatory disorders that associated with chronic renal diseases^23^.

General female subjects with increased baseline PBF over the optimal cut-off values had increased likelihood for predicting the future CKD in our study. Sex difference in adipose tissue might be multifactorial. Females experienced a continuous increase in PBF throughout development and they had higher PBF than males during puberty^24^. Leptin was primarily produced by adipose tissue with circulating levels being positively correlated with total body fat^25^. Hellstrom *et al*. reported the gender difference in circulating leptin concentrations that females had higher levels than males^26^. Renal function decline was caused by increased leptin via triggering a paracrine interaction in proliferation of glomerular endothelial cells, exerting sympathetic nervous activity, and inducing reactive oxygen species^17^.

The strengths of our study are a large population-based survey, appropriate renal function measurement for the study sample, and a cohort analysis for the association between PBF and risks of the future CKD. However, there are several potential limitations among our study. First, the dataset was derived from only an Asian population. Therefore, the limited ethnicity diversity in the participants might not reflect the interaction in terms of racial differences. Second, the measurement of body composition among the study was used by BIA but not by DEXA, the standard method for measuring body fat and muscle mass in general. Next, the biological mechanism through which PBF acted on renal function were not well elucidated. Further researches into the potential underpinnings of the relationship were needed. Last, the information about menopause and postmenopausal years of female participants was unavailable in our study. Sex hormones strongly influence body fat distribution and adipocyte differentiation^27^. Previous studies have reported that menopause-related changes in body fat distribution had risk of cardiometabolic diseases during postmenopausal years^28^. Decrease in estrogen secretion is considered to have a significant effect of obesity in menopausal females^29^.

## Conclusion

Our findings demonstrated the association between the changes of PBF and the decline of renal function in adult population in Taiwan. PBF might be used to predict the risk of the future CKD, particularly in females. Measurement of body fat might provide as a useful tool for surveillance of renal function decline in adult population.

## Methods

### Study design

**Figure 1:**
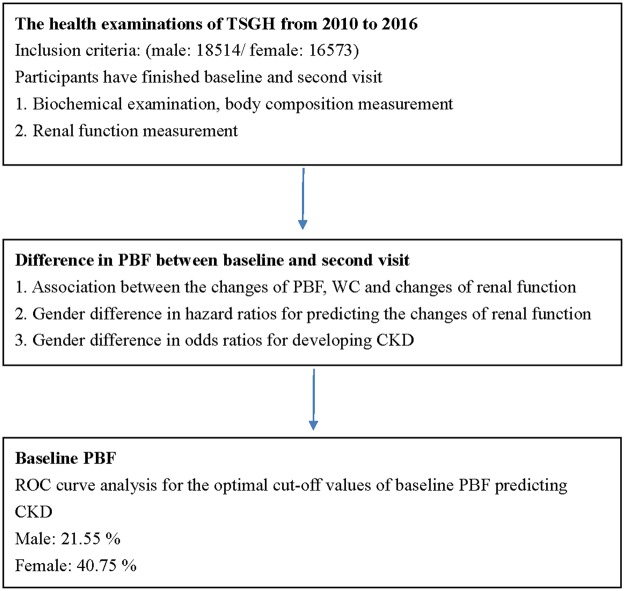
Flow chart which represented the steps of analysis performed in the study.

The present study was performed in the health examinations of Tri-Service General Hospital (TSGH) from 2010 to 2016. Study approval was conduct by the Institutional Review Board (IRB) of TSGH. The TSGH IRB waived the need to obtain individual informed consent because these data were analyzed anonymously. All methods were performed in accordance with the relevant guidelines and regulations of TSGH IRB. The flow chart of the study was shown in Fig. 1. participants who finished biochemical examination, body composition measurement, and renal function measurement at baseline and second visit were included (male: 18514/female: 16573).

### Measurement of renal function

Previous studies had indicated that eGFR using the Modification of Diet in Renal Disease (MDRD) or Chronic Kidney Disease Epidemiology Collaboration (CKD-EPI) equations might not be accurate for Asians^30,31^. Thus, specialists in Japan, China and Thailand subsequently presented different estimations suitable for their citizens^32–34^. In our study, eGFR was estimated by Taiwanese MDRD equation, reported by Chen *et al*., which was better than other renal function equations for Taiwanese adults^13^. The formula of Taiwanese MDRD equation was 1.3096 X MDRD^0.912^. Serum creatinine (Cr) was measured by the uncompensated Jaffe method with the alkaline picrate kinetic test.

### Diagnosis of chronic kidney disease

According to the definition of the Kidney Disease Outcomes Quality Initiative (KDOQI), individuals with a GFR <60 ml/min/1.73 m^2^ for 3 months were identified as having CKD, irrespective of the presence or absence of kidney damage^35^. Markers of kidney damage included: hematuria, electrolyte abnormalities, structural abnormalities detected by imaging^36^.

### Measurement of body composition

BMI was generally used as an attempt to quantify the amount of tissue mass in an individual and a standard for recording obesity^37^. BMI was estimated based on a general formula that the weight of the in kilograms divided by the square of the height in meters (kg/m^2^) of a participant (kg/m^2^). WC was measured at mid-level between the iliac crest and the lower border of the 12^th^ rib. Bioelectrical impedance analysis (BIA) was an effective and valid method for assessing body composition^38^. It was an alternative to more invasive and expensive methods like dual-energy X-ray absorptiometry, computerized tomography, and magnetic resonance imaging. In the present study, we detected PBF by using BIA (InBody720, Biospace, Inc., Cerritos, CA, USA).

### Covariates measurement

Biochemical data were collected by drawing blood samples from subjects after fasting for at least 8 hours. Fasting plasma glucose (FPG) was detected using a glucose oxidase method. Aspartate transaminase (AST) was measured by an enzymatic colorimetric method. The latex-enhanced nephelometry was used to detect high sensitivity C-reactive protein (hsCRP). Uric acid (UA) was measured by the Hitachi 737 automated multichannel chemistry analyzer (Boehringer Mannheim Diagnostics, Indianapolis, IN, USA). Thyroid stimulating hormone (TSH) was accessed by an immune-enzymatic assay. High density lipoprotein cholesterol (HDL-C) were analyzed by using an enzymatic colorimetric method. All experimental methods were performed in accordance with the relevant guidelines and regulations of TSGH.

### Statistical analysis

Statistical estimations used in the study were performed by the Statistical Package for the Social Sciences, version18.0 (SPSS Inc., Chicago, IL, USA) for Windows. The differences between males and females in terms of demographic information and biochemistry data were examined by Student’s t test and Pearson’s chi-square test. A two-sided *p*-value of ≤0.05 was regarded as the threshold for statistical significance. A ROC curve was used to calculate the scores of baseline PBF to predict the presence of CKD, including gender specific cut-off values, AUROC and the corresponding 95%CI. Extend-model approach was performed in the study with multivariable adjustment for pertinent clinical variables as follows: Model 1 included age, gender, and BMI; Model 2 included Model 1 plus proteinuria, UA, AST, albumin, TSH, hsCRP, FPG, and HDL-C; Model 3 included Model 2 plus history of smoking, hypertension (HTN), and diabetes mellitus (DM). A multivariable linear regression model was performed for the association between the changes of PBF and WC with the changes of renal function. A proportional Cox hazard regression model was conducted for the changes of PBF and WC to predict the incident changes of eGFR during the follow-up. A multivariable logistic regression was used for the associations between cut-off values of baseline PBF and the future CKD.
